# Characterization of Five Novel Anti-MRSA Compounds Identified Using a Whole-Animal *Caenorhabditis elegans*/*Galleria mellonella* Sequential-Screening Approach

**DOI:** 10.3390/antibiotics9080449

**Published:** 2020-07-27

**Authors:** Rajamohammed Khader, Nagendran Tharmalingam, Biswajit Mishra, LewisOscar Felix, Frederick M. Ausubel, Michael J. Kelso, Eleftherios Mylonakis

**Affiliations:** 1 Infectious Diseases Division, Department of Medicine, Warren Alpert Medical School of Brown University, Rhode Island Hospital, Providence, RI 02903, USA; rajamohammed_khader@brown.edu (R.K.); nagendran_tharmaligam@brown.edu (N.T.); biswajit_mishra@brown.edu (B.M.); lewis_oscar_felix_raj_lucas@brown.edu (L.F.); 2 Department of Molecular Biology, Massachusetts General Hospital, Boston, MA 02114, USA; ausubel@molbio.mgh.harvard.edu; 3 Department of Genetics, Harvard Medical School, Boston, MA 02115, USA; 4 Molecular Horizons and School of Chemistry and Molecular Bioscience, University of Wollongong, Northfields Avenue, Wollongong 2522, Australia; mkelso@uow.edu.au; 5 Illawarra Health and Medical Research Institute, University of Wollongong, Northfields Avenue, Wollongong 2522, Australia

**Keywords:** antibiotic, high-throughput screening (HTS), MRSA, MIC, *Caenorhabditis elegans*, *Galleria mellonella*

## Abstract

There is a significant need to combat the growing challenge of antibacterial drug resistance. We have previously developed a whole-animal dual-screening platform that first used the nematode *Caenorhabditis elegans*, to identify low-toxicity antibacterial hits in a high-throughput format. The hits were then evaluated in the wax moth caterpillar *Galleria mellonella* infection model to confirm efficacy and low toxicity at a whole animal level. This multi-host approach is a powerful tool for revealing compounds that show antibacterial effects and relatively low toxicity at the whole organism level. This paper reports the use of the multi-host approach to identify and validate five new anti-staphylococcal compounds: (1) 4,4′,4″-(4-propyl-[1*H*]-pyrazole-1,3,5-triyl)*tris*phenol(PPT), (2) (1*S*,2*S*)-2-[2-[[3-(1*H*-benzimidazol-2-yl)propyl]methylamino]ethyl]-6-fluoro-1,2,3,4-tetrahydro-1-(1-methylethyl)-2-naphthalenyl cyclopropanecarboxylate dihydrochloride(NNC), (3) 4,5,6,7-tetrabromobenzotriazole (TBB), (4) 3-[2-[2-chloro-4-[3-(2,6-dichlorophenyl)-5-(1-methylethyl)-4-isoxazolyl]methoxy]phenyl]ethenyl] benzoic acid(GW4064), and (5) *N*-(cyclopropylmethoxy)-3,4,5-trifluoro-2-[(4-iodo-2-methylphenyl)amino] benzamide(PD198306). The compounds reduced the severity of methicillin-resistant *Staphylococcus aureus* (MRSA, strain MW2) infections in both *C. elegans* and *G. mellonella* and showed minimal inhibitory concentrations (MICs) in the range of 2–8 µg/mL. Compounds NNC, PPT, and TBB permeabilized MRSA-MW2 cells to SYTOX green, suggesting that they target bacterial membranes. Compound TBB showed synergistic activity with doxycycline and oxacillin against MRSA-MW2, and compounds PPT, NNC, GW4064, and PD198306 synergized with doxycycline, polymyxin-B, gentamicin, and erythromycin, respectively. The study demonstrates the utility of the multi-host approach with follow-up hit characterization for prioritizing anti-MRSA compounds for further evaluation.

## 1. Introduction

*Staphylococcus aureus* isolates that show resistance to methicillin (methicillin-resistant *S. aureus*, MRSA) were first reported in the United Kingdom in 1961, and soon after, in other European countries, Japan, Australia, and the US [1]. According to the Centers for Disease Control and Prevention (CDC), in the US, there are more than 35,000 deaths and 90,000 severe cases of MRSA infection each year [2]. Vancomycin is typically the antibiotic of choice for treating serious Gram-positive bacterial infections, including MRSA, but reports of vancomycin-resistant and vancomycin-intermediate *S. aureus* strains are becoming more common [3].

Whole animal-based high-throughput screening (HTS) is a powerful tool for discovering antibacterial compounds, including those that would otherwise be missed by traditional antibiotic screens that report compounds that inhibit bacterial growth [4]. Our laboratory has developed a fully automated HTS platform that utilizes the nematode *Caenorhabditis elegans* to identify compounds that cure *Staphylococcus aureus* (MRSA) [5], *Enterococcus faecalis* [6], or *Pseudomonas aeruginosa* [7] infections in *C. elegans* without showing frank host toxicity that results in the death of the nematodes. Since these pathogens kill *C. elegans*, hit compounds are those that reduce *C. elegans* killing, resulting in an excess of live worms compared to non-treated pathogen-infected nematodes. Highly toxic compounds do not emerge as hits in the assay, because they kill the nematodes.

Here, we describe the coupling of the *C. elegans* HTS platform with a secondary screen that uses MRSA infection of *Galleria mellonella* (wax moth) larvae (caterpillars) to validate the anti-staphylococcal activity and low whole-animal toxicity of hit compounds at the whole-organism level [8]. In this study, we used the dual-host approach to identify and validate five new anti-MRSA compounds, and report follow-up characterization of their antibacterial and other properties.

## 2. Results

### 2.1. Identification of Compounds That Block the Ability of MRSA to Kill C. elegans

**Figure 1:**
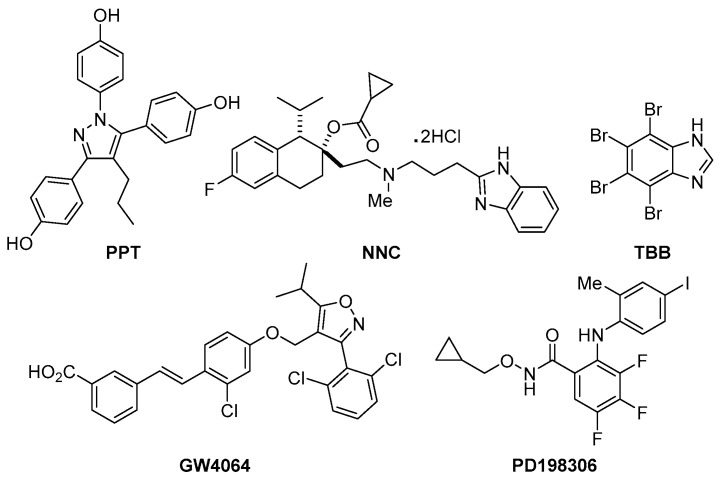
Chemical structures of PPT, NNC, TBB, GW4064, and PD198306.

We previously reported that high throughput screening for compounds that reduce the ability of MRSA to kill *C. elegans* yielded 185 hits from a total of 82,000 unique compounds tested [9]. Among the 185 hits, five compounds that prolonged *C. elegans* survival at a screening concentration of 2.86 µg/mL (PPT, NNC, TBB, GW4064, and PD198306; Figure 1) were chosen for further evaluation, because: (1) previous studies had reported some form of bioactivity for each and (2) their Z-scores in the *C. elegans* screen were higher than 2.0, indicating a robust response (Table 1 and Table 2).

### 2.2. Galleria Mellonella Assays

The five hits were next tested in the *G. mellonella*–MRSA model to validate their activity in a second whole-animal infection assay. Maximum tolerated doses (MTDs) were obtained by challenging uninfected larvae with increasing doses of the compounds (10, 20, and 30 mg/kg). All five compounds exhibited MTDs of 10 mg/kg (data not shown) and this concentration was used in the infection model.

**Figure 2:**
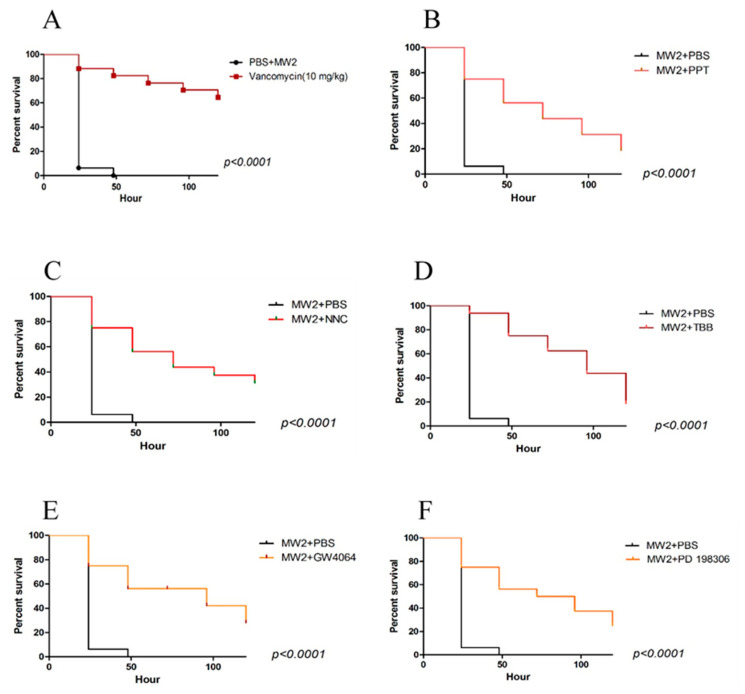
*Galleria mellonella* larvae infected with *S.aureus*-MW2 were treated with vancomycin (10 mg/kg) (**A**) or one of the five compounds (**B**–**F**, 10 mg/kg) and survival was monitored for 120 hr. Statistical analyses (Kaplan–Meier survival analysis with log-rank test) were conducted using GraphPad Prism version 8 (GraphPad Software, La Jolla, CA, USA).

Larvae were infected with MRSA MW2 (5 × 10^5^ CFU) in the left rear proleg, and after two hours, the compounds were introduced separately at 10 mg/kg into the right rear proleg and larvae survival was monitored for 120 h. All compounds prolonged survival relative to untreated controls (Figure 2), although none were as effective as vancomycin at 10 mg/kg. Untreated larvae showed a median post-infection survival of only 1 day, while GW4064, NNC, PD198306, and PPT each extended median survival to 2 days. TBB treatment extended median survival to 3 days.

### 2.3. Antibacterial Susceptibility

Antibacterial activity of the five compounds was evaluated against MRSA-MW2 (Table 3) and four additional clinical *S. aureus* isolates (Table 4). All of the compounds were found to potently inhibit the growth of MW2 and the isolates, with each showing MICs in the range of 2–8 µg/mL. By comparison, the MIC of vancomycin was 1 µg/mL against MW2 and 2 µg/mL against other isolates. The minimum bactericidal concentration (MBC) of PPT against MW2 was 32 µg/mL, and for NNC, TBB, GW4064, and PD198306 it was >64 µg/mL. When tested against ESKAPE pathogens (Table 4), good activity (MIC, 2–8 µg/mL) was seen against the Gram-positive bacterium *Enterococcus faecium*; however, poor activity was observed against Gram-negative pathogens except with PD198306, which showed modest activity (MIC 32 µg/mL) against *Acinetobacter baumanii*.

**Figure 3:**
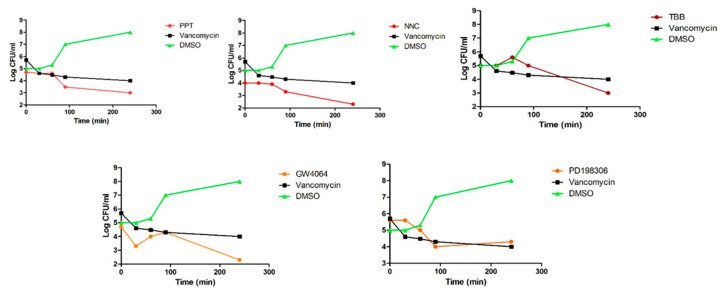
MRSA-MW2 cells were exposed to compounds at 64 µg/mL and cell viability was monitored over 4 hr. DMSO 0.64% and vancomycin (16 µg/mL) were included as controls.

Time-to-kill assays were used to further probe bactericidal/bacteriostatic properties against MRSA MW2 (Figure 3). Antibacterial compounds are generally considered bactericidal, if the MBC is no more than four times higher than the MIC. Bactericidal activity is defined as greater than 3 log_10_ -fold decrease in colony forming units, which is equivalent to 99.9% killing of the inoculum. If the MBC is outside this range, compounds are bacteriostatic [15]. According to these criteria, all five compounds were bacteriostatic.

### 2.4. Membrane Permeabilization

**Figure 4:**
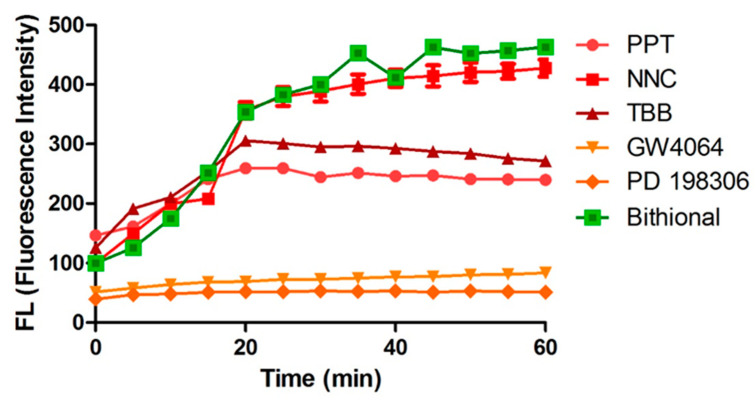
Cellular fluorescence of MRSA-MW2 cells treated with sytox green and test compounds (64 µg/mL) monitored over 1 h. Bithionol (64 µg/mL) was included as positive control.

To determine whether the compounds target bacterial membranes, MRSA MW2 was treated with the compounds and uptake of the membrane-impermeable DNA-binding fluorescent dye Sytox green was monitored over 1 h (Figure 4). Exposure of cells to the compounds at 64 µg/mL revealed that TBB, PPT, and NNC caused increases in cellular fluorescence, suggesting that they damage the membrane. Compounds GW4064 and PD198306 showed no changes in cellular fluorescence, suggesting that they do not elicit antibacterial action through effects on membranes.

### 2.5. Human Red Blood Cell Hemolysis Assays and Cytotoxicity

**Figure 5:**
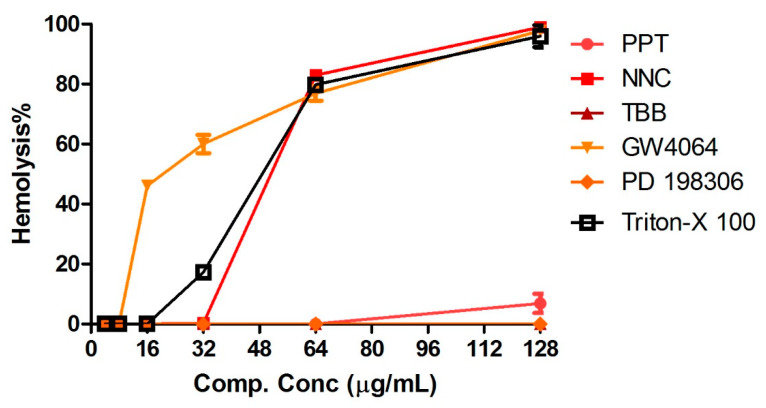
Human red blood cells (RBCs) (2%) were exposed to two-fold serial dilutions of compounds and hemolysis was measured after 1 hr. A sample treated with 1% Triton-X 100, which causes 100% hemolysis, was used as the positive control.

**Figure 6:**
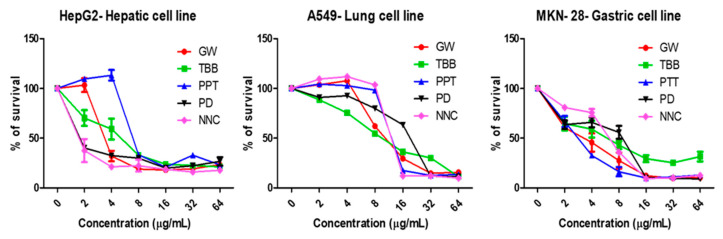
Cytotoxicity: Mammalian cells (HepG2, A549, and MKN-28) were treated with 2-fold serial dilutions of compounds and the cytotoxicity was measured after 24 hr.

Serial dilutions of the compounds (2–128 µg/mL) were added to human red blood cells (RBCs) and monitored for hemolytic activity (Figure 5). NNC and GW4064 showed hemolysis at concentrations of 32–64 µg/mL and 8-16 µg/mL, respectively, and PPT caused slight hemolysis at 128 µg/mL. PD198306 and TBB showed no hemolytic activity. Eukaryotic cell cytotoxicity of the compounds was tested using the MTT (3-(4,5-dimethylthiazol-2-yl)-2,5-diphenyltetrazolium bromide) method with liver (HepG2), lung (A549), and gastric (MKN-28) cell lines (Figure 6 and Table 5). All of the compounds were found to be relatively toxic in these cell-based assays, inhibiting proliferation at concentrations close to their *S. aureus* MICs.

### 2.6. Antibacterial Synergy

The combination of two antibiotics with different modes of action can reduce bacterial resistance and sometimes even restore clinical efficacy of an antibiotic that has lost effectiveness [16]. The five compounds were tested against MRSA-MW2 in the presence of five clinical antibiotics from different classes (i.e., polymyxin B, gentamycin, erythromycin, doxycycline, and oxacillin). Paired combinations of compounds and their FIC indices are listed in (Table 6). Synergic effects, where the combined antibacterial activity of the two compounds is greater than the sum of the individual compound activities, are identified by FIC index ≤ 0.5, antagonism by FIC index > 4.0, and no interaction by 0.5 < FIC index < 4.0. Polymyxin-B showed synergistic activity with NNC and gentamicin synergized with GW4064. Erythromycin showed synergy with PD198306. Doxycycline and oxacillin both synergized with TBB, but only doxycycline with PPT. None of the compounds antagonized the activity of the antibiotics.

## 3. Discussion

The aim of this study was to characterize the antibacterial properties of five synthetic compounds with activity against MRSA. The activity of the compounds against MRSA was originally identified using a *C. elegans–*MRSA whole-animal infection model. In this study, compound activities were confirmed at the whole-animal level using the *G. mellonella*–MRSA model [5]. The *G. mellonella* infection model is a quick and inexpensive way to confirm the efficacy of hit compounds against *S. aureus* in vivo [17], but is too cumbersome and labor intensive to implement for large scale screening of chemical libraries. Hence, we used the *G. mellonella* model for follow-up validation of *C. elegans* high throughput screening hits to enable prioritization of compounds for further characterization.

The five compounds chosen for study here (PPT, NNC, TBB, GW4064, and PD198306) all prolonged the survival of both *C. elegans* and G. *mellonella* infected with MRSA. A literature survey revealed that although the compounds had reported bioactivities, none had previously been reported to have antibacterial activity. PPT is described as a subtype-selective ERα agonist. We found that the MIC of PPT against MRSA-MW2 and four clinical isolates was 8 µg/mL. The compound showed potent membrane permeabilizing properties and some hemolytic activity in human red blood cells, suggesting it targets the bacterial membrane. Synergy was observed with PPT and the antibiotic doxycycline. As a class, pyrazoles show a wide range of biological activities, including antimicrobial, antifungal, antitubercular, anti-inflammatory, anticancer, antiviral, neuroprotective, and estrogen receptor (ER) activity [10]. Some pyrazole derivatives have been shown to exhibit activity against both Gram positive and Gram-negative bacteria and an *N*-phenyl-1*H*-pyrazole-4-carboxamide derivative increased the survival of *G. mellonella* infected with *S. aureus* [18]. PPT is a highly hydrophobic pyrazole containing three phenolic substituents and it is possible that these properties are responsible for the significant toxicity observed against eukaryotic cells.

NNC is a reported calcium channel blocker [11]. The compound showed a MIC of 8 µg/mL against MRSA MW2 and was slightly more potent against the other four isolates. As with PPT, NNC appears to target the bacterial membrane, since it permeabilizes MRSA cells and shows hemolytic activity at 32 µg/mL. It was also found to be cytotoxic against all three eukaryotic cell lines.

TBB is a reported inhibitor of protein kinase 2 (CK2) [12]. The compound showed a MIC between 4–6 µg/mL against MRSA and the clinical isolates and while it appears to be membrane active against MRSA-MW2, no red blood cell hemolysis was observed. The compound synergized with doxycycline and oxacillin against MRSA, however, like PPT and NNC, it was also toxic to eukaryotic cell lines at concentrations around its MIC.

GW4064 is a reported farnesoid X receptor (FXR) agonist [19] that suppresses cell proliferation in several cancer lines, but not normal cells [20]. The compound showed a MIC of 4–8 µg/mL against the MRSA strains and synergized with gentamycin. While GW4064 appears not to be membrane active against MRSA MW2, it showed hemolytic activity at 8 µg/mL and was cytotoxic to eukaryotic cells.

PD198306 is a potent and selective non-ATP competitive inhibitor of MEK1/2 that shows antihyperalgesic properties [14]. The compound showed the lowest MIC (2 µg/mL) against MRSA MW2, which was similar for the four other isolates. Its activity was bacteriostatic and the compound synergized with erythromycin. Importantly, PD198306 does not appear to be membrane active against MRSA and shows no hemolytic activity, suggesting that its antibacterial action may be more target-specific than the other compounds. However, PD198306 was also cytotoxic towards the three eukaryotic cell lines at concentrations around its MIC, potentially reducing its attractiveness for further study.

In summary, this study demonstrates the utility of the *C. elegans*/*G. mellonella* whole animal dual-screening approach for identifying compounds of interest for study as new agents against MRSA. The sequential use of two independent model hosts is a powerful tool for prioritizing compounds, as it confirms that efficacious anti-microbial compounds identified by HTS in *C. elegans* are indeed relatively non-toxic at a whole animal level and may warrant further study in vertebrate animals. Although none of the five compounds exhibited a high level of toxicity at the whole animal level, all five compounds did inhibit the proliferation of human cells at concentrations approximating the MICs for MRSA. Despite this, the chemical scaffolds, particularly PD198306, represent good starting points for traditional medicinal chemistry aimed at decreasing toxicity while maintaining antimicrobial activity.

## 4. Materials and Methods

### 4.1. Hit Compounds

The five compounds: (4,4′,4″-(4-propyl-[1*H*]-pyrazole-1,3,5-triyl)*tris*phenol, PPT; (1*S*,2*S*)-2-[2-[[3-(1*H*-benzimidazol-2-yl)propyl]methylamino]ethyl]-6-fluoro-1,2,3,4-tetrahydro-1-(1-methylethyl)-2- naphthalenyl cyclopropanecarboxylate dihydrochloride, NNC; 4,5,6,7-tetrabromobenzotriazole, TBB; 3-[2-[2-chloro-4-[3-(2,6-dichlorophenyl)-5-(1-methylethyl)-4-isoxazolyl]methoxy]phenyl] ethenyl] benzoic acid, GW4064; and *N*-(cyclopropylmethoxy)-3,4,5-trifluoro-2-[(4-iodo-2-methylphenyl)amino] benzamide, PD198306) were purchased from Tocris Bioscience at the following levels of purity: PPT: >99.7%, NNC: >98%, TBB: >99%, GW4064: >97%, and PD198306: 98.8%. These compounds are members of a set of 185 hits identified previously in a *C. elegans*–MRSA-MW2 screen of 82,000 synthetic, low molecular weight compounds that block the ability of MRSA to kill nematodes [5,9]. Hits were identified based on Z-scores. Z-scores are calculated from the ratio of alive versus dead worms after treatment with test compounds and represents the number of standard deviations (SD) the test compound differs from the mean using the formula *Z* = (*x* − *μ*)/*σ*; where *x* is the raw sample score, *μ* is the mean of the population and *σ* is the standard deviation of the population. Samples with *Z* > 2*σ* are considered hits [5].

### 4.2. Galleria Mellonella Survival Assays

Assays were performed as described previously [21]. Briefly, sixteen randomly selected *G. mellonella* larvae (Vanderhorst, Inc., St. Mary’s, OH, USA) between 300–350 mg were used for each test group. MRSA-MW2 was grown in Tryptic Soy Broth (TSB), washed with phosphate buffered saline (PBS, pH = 7.4), and diluted to OD_600_ = 0.3 before inoculation into *G. mellonella* larvae. A 10-μL inoculum was injected into the last left proleg using a 10-μL Hamilton syringe. After 2 h, compounds were administered into the last right proleg and larvae were incubated at 37 °C. *G.mellonella* survival was monitored for 120 h, and death was confirmed when there was no response. Killing curves and differences in survival were analyzed by the Kaplan–Meier method using GraphPad Prism version 8 (GraphPad Software, La Jolla, CA, USA). Statistical analysis (Kruskal–Wallis test) was carried out using the same program.

### 4.3. Antibacterial Susceptibility Assays

In vitro antibacterial activities were determined using the broth microdilution method [22]. Assays were performed as described in Clinical and Laboratory Standards Institute protocols in triplicate using cation-adjusted Muller–Hinton broth (Becton Dickinson and Company, Franklin Lakes, NJ, USA) in 96-well plates with a total assay volume of 100 µL. Two-fold serial dilutions of test compounds were prepared over the concentration range of 1–64 µg/mL. The bacterial concentration was adjusted to OD_600_ = 0.06. MW2 was incubated with the test compounds at 37 °C for 18 h, OD_600_ was measured and the lowest concentration of each compound that visually inhibited bacterial growth was reported as the minimal inhibitory concentration (MIC) [23]. A 10 µL aliquot from each well in the MIC assays was plated onto Muller–Hinton agar (Becton Dickinson and Company, Franklin Lakes, NJ, USA) and incubated overnight at 37 °C. The lowest concentration of compound for which no growth was observed on agar plates was reported as the minimal bactericidal concentration (MBC).

### 4.4. Time-To-Kill Assays

Time-to-kill assays were performed as previously described [24]. Briefly, overnight cultures of MRSA-MW2 were diluted in fresh Tryptic Soy Broth (TSB) to a density of 10^8^ cells/mL and placed into 10 mL tubes (Becton Dickinson and Company, Franklin Lakes, NJ, USA). Test compounds dissolved in DMSO over the concentration range 4–64 µg/mL were added and the tubes incubated at 37 °C. At periodic intervals, aliquots from each tube were serially diluted with TSB and plated onto tryptic soy agar (TSA) (Becton Dickinson and Company, Franklin Lakes, NJ, USA). CFUs were enumerated after overnight incubation at 37 °C. Assays were carried out in triplicate.

### 4.5. Membrane Permeabilization

Sytox Green (Life Technologies, Needham, MA, USA) membrane permeabilization assays were performed as previously described [25]. The experiments were carried out in duplicate in 96-well plates. Cells were grown to log phase and were harvested by centrifuging at 3700× *g* for 5 min. The cells were washed twice with PBS and resuspended to OD_600_ = 0.2, following which 5 µM Sytox green was added to the cells and the bacteria-dye mixture were incubated in the dark for 30 mins.

Cell suspensions (50 µL) were added to 50 µL of test compounds (64 µg/mL in PBS) and fluorescence intensity was measured (excitation 485 nm, emission 530 nm) periodically over 60 min. Bithionol, which our laboratory previously showed permeabilizes MRSA [26], was used as a positive control. Membrane damage caused by compounds was indicated by increases in cellular fluorescence caused by enhanced permeability of the DNA staining, membrane-impermeable dye.

### 4.6. Human Red Blood Cell (RBC) Hemolysis Assays

Human erythrocytes (Rockland Immunochemicals, Limerick, PA, USA) were used to measure hemolytic activity of the compounds, as previously described [27]. Human erythrocytes (4% in PBS, 50 µL) were added to 50 µL of test compounds serially diluted in PBS, with Triton X-100 and PBS serving as a positive and negative controls, respectively. After incubation at 37 °C for 1 hr, the plates were centrifuged at 500× *g* for 5 min and 50 µL of the supernatant was transferred from each well to a second 96-well plate and the absorbance at 540 nm was measured. Assays were carried out in triplicate.

### 4.7. Cytotoxicity Assays

Mammalian cell lines HepG2 (hepatic cells), A549 (epithelial cells), and MKN-28 (gastric cells) were used to measure the anti-proliferative properties of test compounds, as described elsewhere [28]. Cells were grown in Dulbecco’s Modified Eagle Medium (DMEM) (Gibco, Waltham, MA, USA) supplemented with 10% Fetal Bovine Serum (FBS) (Gibco, Waltham, MA, USA) and 1% penicillin/streptomycin (Gibco, Waltham, MA, USA) and maintained at 37 °C in 5% CO_2_. After harvesting, the cells were suspended in DMEM and 100 µL was added to the wells of 96-well plates. The test compounds were serially diluted in serum and antibiotic free DMEM, added to the monolayer of cells, and incubated at 37 °C in 5% CO_2_ for 24 hr. Percentage survival was calculated by comparison to DMSO-treated vehicle controls. Assays were carried out in triplicate.

### 4.8. Checkboard Assays

Antibacterial synergy for combinations of the compounds with each other and with clinical antibiotics (doxycycline, gentamicin, polymyxin-B, oxacillin, and erythromycin) was measured using checkerboard assays. In short, Cultures of MRSA-MW2 were adjusted to OD_600_ = 0.06 and one compound was arrayed horizontally and other vertically in 96 well plates. The assays were carried out in triplicate, exactly as described for the antibacterial susceptibility assays. The inhibitory effects of compound combinations were evaluated using the Fractional Inhibitory Concentration (FIC) index, derived from the formula:

FIC index = FIC_A_ + FIC_B_ = [A]/MIC_A_ + [B]/MIC_B_

MIC of substance A tested in combination /MIC of substance A tested alone + MIC of substance B tested in combination/MIC of substance B tested alone. Synergy is indicated when FIC ≤ 0.5, indifference when 0.5 ≤ FIC ≤ 4, and antagonism when FIC > 4. Experiments were performed in duplicate and report the average FIC index [29].

## Figures and Tables

**Table 1:** List of compound names and reported bioactivities.

Compounds	Chemical Name	Reported Bioactivity
PPT	4,4′,4″-(4-Propyl-[1*H*]-pyrazole-1,3,5-triyl)*tris*phenol	Prevents ovariectomy-induced weight gain and loss of bone mineral density, and induces gene expression in the hypothalamus following systemic administration in vivo [10].
NNC	(1*S*,2*S*)-2-[2-[[3-(1*H*-Benzimidazol-2-yl)propyl]methylamino]ethyl]-6-fluoro-1,2,3,4-tetrahydro-1-(1-methylethyl)-2-naphthalenyl cyclopropanecarboxylate dihydrochloride	Highly selective T-type calcium channel blocker. Displays IC_50_ values of 6.8 and >100 μM for inhibition of Ca_v_3.1 T-type channels and HVA currents respectively in INS-1 cells [11].
TBB	4,5,6,7-Tetrabromobenzotriazole	Cell-permeable, selective inhibitor of casein kinase-2.Exhibits modest discrimination between CK2 subunits [12].
GW4064	3-[2-[2-Chloro-4-[[3-(2,6-dichlorophenyl)-5-(1-methylethyl)-4-isoxazolyl]methoxy]phenyl]ethenyl]benzoic acid	Selective, non-steroidal farnesoid X receptor (FXR) agonist Shown to suppress autophagy in nutrient-deprived mouse hepatocytes [13].
PD198306	*N*-(Cyclopropylmethoxy)-3,4,5-trifluoro-2-[(4-iodo-2-methylphenyl)amino]-benzamide	Potent inhibitor of MEK1/2.Highly selective for MEK.Antihyperalgesic; blocks static allodynia in the streptozocin model of neuropathic pain [14].

**Table 2:** Z-scores of *C. elegans* screening hits.

Compound	*Z*-Score
PPT	5.54
NNC	5.83
TBB	3.16
GW4064	3.28
PD198306	3.43

**Table 3:** Antibacterial activity (µg/mL) of compounds against MRSA MW2.

Compounds	MIC	MBC
PPT	8	32
NNC	8	>64
TBB	6	>64
GW4064	8	>64
PD198306	2	>64
Vancomycin	1	8

**Table 4:** Antibacterial activity of compounds against clinical *S. aureus* isolates and ESKAPE pathogens.

Clinical *S. aureus* Isolates and ESKAPE Pathogens	MIC (µg/mL)					
	PPT	NNC	TBB	GW4064	PD198306	Vancomycin
*BF1*	8	4	4	4	2	2
*BF2*	8	2	4	4	2	2
*BF3*	8	4	8	8	2	2
*BF4*	8	4	4	8	2	2
*Enterococcus faecium*	8	8	4	8	2	4
*Klebsiella pneumoniae*	>64	>64	>64	>64	>64	>64
*Acinetobacter baumannii*	>64	>64	>64	>64	32	>64
*Enterobactor aerogens*	>64	>64	>64	>64	>64	>64
*Pseudomonas aeruginosa*	>64	>64	>64	>64	>64	>64

**Table 5:** Eukaryotic cell cytotoxicity of compounds (µg/mL).

Compounds	HepG2	A549	MKN-28
PPT	8	16	8
NNC	2	16	8
TBB	8	16	16
GW4064	4	16	8
PD198306	2	16	16

**Table 6:** Fractional Inhibitory Concentration (FIC) Index of test compounds with selected antibiotics from different classes.

Compounds	Polymyxin B	Gentamycin	Erythromycin	Doxycycline	Oxacillin
PPT	2.0	1.0	0.75	0.5	1.0
NNC	0.5	0.75	1.0	0.75	2.0
TBB	2.0	1.0	0.625	0.5	0.5
GW4064	0.75	0.5	1.0	2.06	1.0
PD198306	1.0	0.625	0.5	2.0	0.75
